# Serotonergic antidepressants are associated with increased bleeding events within 30-days after total shoulder arthroplasty: a propensity-matched analysis of 54,291 patients

**DOI:** 10.1007/s00402-026-06254-y

**Published:** 2026-03-12

**Authors:** John T. Strony, Andrew J. Moyal, Jeremy M. Adelstein, Robert J. Burkhart, Anthony M. Imbrogno, Rayyan Abid, Robert J. Gillespie, Raymond E. Chen

**Affiliations:** 1 University Hospitals Drusinsky Sports Medicine Institute, Cleveland, USA; 2 Case Western Reserve University School of Medicine, Cleveland, USA

**Keywords:** Total shoulder arthroplasty, SNRIs, SSRIs, Postoperative outcomes, Complications

## Abstract

**Introduction:**

Selective serotonin reuptake inhibitors (SSRIs) and serotonin-norepinephrine reuptake inhibitors (SNRIs) are associated with bleeding events following orthopaedic surgery. However, their effect on total shoulder arthroplasty (TSA) outcomes is unknown.

**Methods:**

Patients undergoing primary anatomic or reverse TSA in the United States were identified in the TriNetX database using Current Procedural Terminology (CPT) and International Classification of Disease (ICD-10) codes. TSA for fracture was excluded. Two cohorts were created based on preoperative serotonergic antidepressant use. Cohorts were matched to 14 demographic, comorbidity, laboratory and medication parameters. Outcomes were analyzed at one week, one month and three months postoperatively. Significance was set at *p* < 0.05.

**Results:**

7,374 matched patients were included per cohort. The SSRI/SNRI cohort had significantly higher odds for post-hemorrhagic anemia and transfusion on the day of surgery (Anemia - OR 1.50, 95%CI 1.28–1.76; Transfusion - OR 2.55, 95%CI 1.27–5.13), at seven-days post-op (Anemia - OR 2.78, 95%CI 1.68–4.58; Transfusion - OR 2.58, 95%CI 1.39–4.79) and at one month post-op (Anemia - OR 2.73, 95%CI 1.37–5.46; Transfusion - OR 2.31, 95%CI 1.09–4.88). From one through three-months post-operatively, the two cohorts did not differ in rates of postoperative anemia, hematoma/hemorrhage, or reoperation.

**Conclusions:**

Serotonergic antidepressants were associated with a higher rate of bleeding events within 30 days postoperatively after TSA. These results are seen even when propensity matching for PT/PTT and platelet function. Complications normalize after 30 days and do not appear to pose a long-term risk.

**Supplementary Information:**

The online version contains supplementary material available at 10.1007/s00402-026-06254-y.

## Introduction

Total shoulder arthroplasty, including reverse and anatomic total shoulder arthroplasty (TSA), is a versatile procedure that is used to treat an array of shoulder pathology, including glenohumeral osteoarthritis, avascular necrosis, proximal humerus fractures, massive irreparable rotator cuff tears, and cuff tear arthropathy [1–11].Predictably, as the indications have expanded, the prevalence of total shoulder arthroplasty continues to rise in the United States [7, 12–14].

Selective serotonin reuptake inhibitors (SSRIs) and serotonin norepinephrine reuptake inhibitors (SNRIs) are routinely prescribed because of their efficacy and safety in treating numerous psychiatric conditions, including but not limited to major depressive disorder and generalized anxiety disorder [15–20]. From 2015 to 2018, 13.2% of adults aged 18 years or older used antidepressant medication and the prevalence of antidepressant use increased with increasing age [21]. Although these serotonergic antidepressants are relatively safe, they have been associated with significant adverse reactions, the most pertinent of which are adverse bleeding events [22–25]. This phenomenon is thought to occur because SSRI and SNRIs decrease the concentration of serotonin within the platelet dense granules which, in turn, impairs platelet aggregation [26].

Prior studies have shown that the use of SSRI or SNRIs during the perioperative period is associated with higher rates of bleeding events postoperatively [27–32]. However, to the best of our knowledge, no such study has been performed in the context of TSA and, therefore, their effect on total shoulder arthroplasty (TSA) outcomes is unknown. Therefore, the purpose of this study was to determine the association between perioperative serotonergic antidepressant medications and immediate- and short-term outcomes following TSA. We hypothesized that serotonergic antidepressant medications would be associated with worse outcomes, regardless of time point, following TSA.

## Methods

### Patient selection and study design

The TriNetX Global Collaborative network includes aggregated and deidentified data from approximately 142 million patient electronic medical records (EMR). The TriNetX platform follows Health Insurance Portability and Accountability Act (HIPAA) and General Data Protection Regulation (GDPR) guidelines for patient de-identification. Thus, this study has been deemed as non-human subject research by our institutional review board.

On March 6th, 2024, patients undergoing primary anatomic or reverse TSA were identified in the TriNetX database using Current Procedural Terminology (CPT) and International Classification of Disease, 10th Revision (ICD-10) codes (Supplement 1). Patients from 66 participating health care organizations in the United States were included. Patients were excluded if they underwent hemiarthroplasty of the shoulder or if they underwent TSA for definitive management of an acute proximal humerus fracture or malunion/nonunion. Patients with a prior history of non-shoulder arthroplasty (e.g., hemiarthroplasty of the hip, total ankle arthroplasty [TAA], total knee arthroplasty [TKA]) were also excluded. Additionally, any patient with a prior history of knee, hip, or other prosthetic joint infection were excluded. Identified patients were classified into one of two possible cohorts. Cohort 1 consisted of patients who underwent TSA who were taking either an SSRI or an SNRI (Supplement 2) within 1 day of the index procedure. This includes patients with an active prescription on the day before index surgery but does not necessarily imply day-of ingestion. Information regarding dosage, adherence, and duration of medication are not provided by the TriNetX database, so chronicity was not assessed. Studies medications included citalopram, escitalopram, fluoxetine, fluvoxamine, paroxetine, sertraline, venlafaxine, desvenlafaxine, duloxetine, milnacipran, and levomilnacipran. Cohort 2 consisted of patients who underwent TSA who had no history of SSRI or SNRI consumption.

**Fig. 1:**
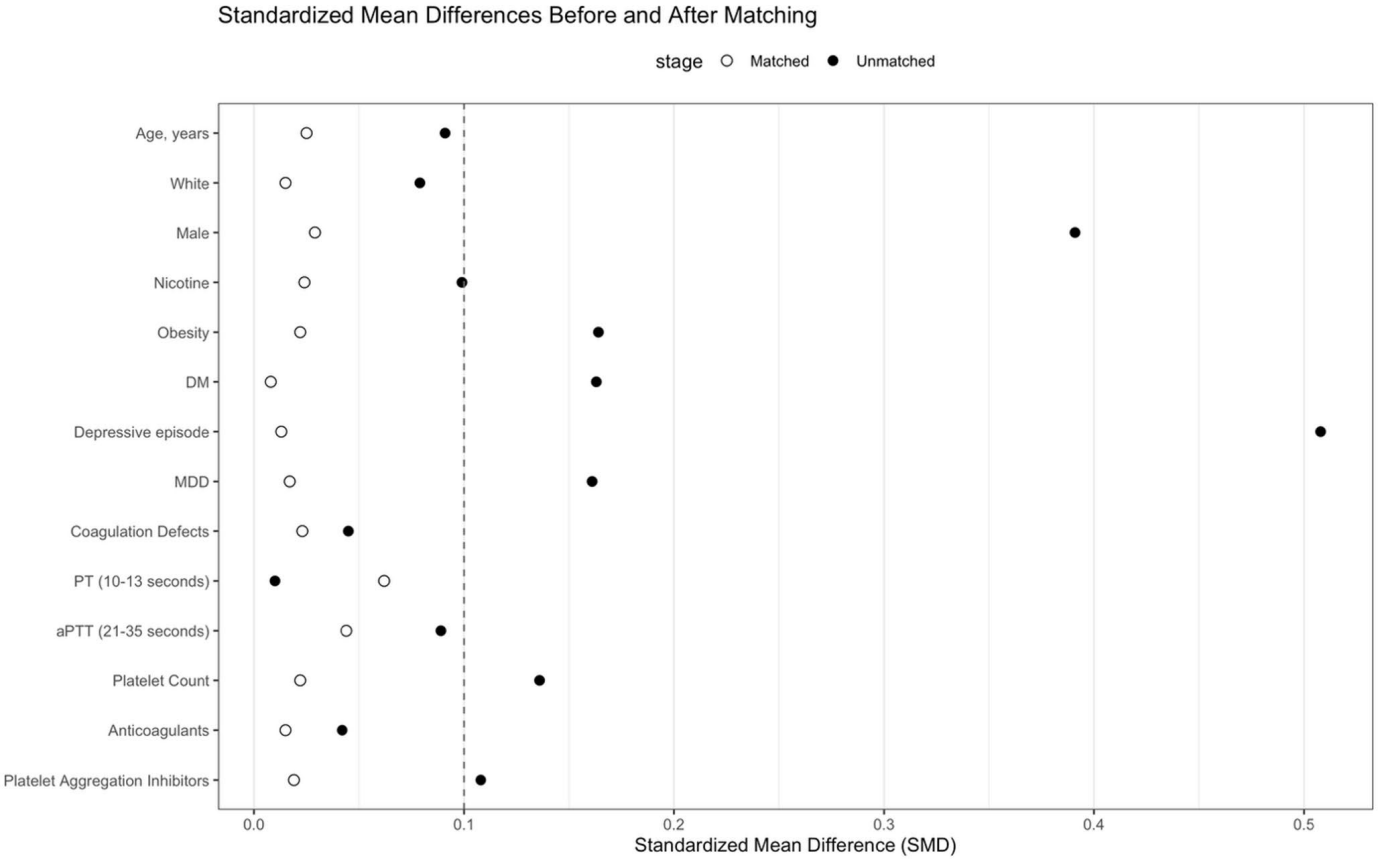
Love plot demonstrating standardized mean differences for variables before and after matching

Data regarding demographics, comorbidities, laboratory parameters and medications were collected. Specific variables of interest included age at index procedure, race/ethnicity, sex, nicotine use, obesity, diabetes mellitus, depression, coagulation defects, prothrombin time (PT), activated partial thromboplastin time (aPTT), platelet count, anti-coagulant prescriptions and platelet aggregation inhibitor prescriptions. All variables were identified to occur within one month of surgery. To eliminate confounding effects of these specific variables, a propensity match occurred ensuring no statistical differences between the 14 variables provided. Patients with missing laboratory values or medications were excluded from the propensity match. Propensity matching was performed using 1:1 matching using a nearest neighbor greedy matching algorithm without replacement based on a logit regression model with a caliper of 0.25 times the standard deviation. The balance threshold for SMD was less than 0.10. Exact matching was used for categorical variables (Fig. 1).

Outcomes were assessed for the day of surgery, post-operative days 1–7, post-operative days 7–30, and post-operative days 30–90. Specific outcomes included emergency department (ED) utilization, intensive care unit (ICU) utilization, hospital readmissions, need for ventilation, post-hemorrhagic anemia, hematoma/hemorrhage, transfusion, wound dehiscence, venous-thromboembolism, shoulder reoperation shoulder irrigation and debridement and mortality (Supplement 3). The primary outcome of interest included bleeding events including post-hemorrhagic anemia, hematoma/hemorrhage, and transfusions. Outcomes occurred following index procedure but were not necessarily linked to TSA. To maintain HIPAA compliance, all patient counts of < 10 are reported as 10 patients within the TriNetX platform.

### Data analysis

Data analysis was completed utilizing the TriNetX statistical package version 7.0.322. Mean and standard deviations (SD) were calculated for continuous variables and assessed with Mann-Whitney U testing, while frequencies and percentages were calculated for categorical variables and assessed with chi-squared tests. Comparisons of primary and secondary outcomes were then made on balanced cohorts using odds ratio (OR). Post-match ORs were calculated using a logistic regression function within the TriNetX platform. Statistical significance was set at *p* < 0.05.

## Results

### Patient characteristics

**Fig. 2:**
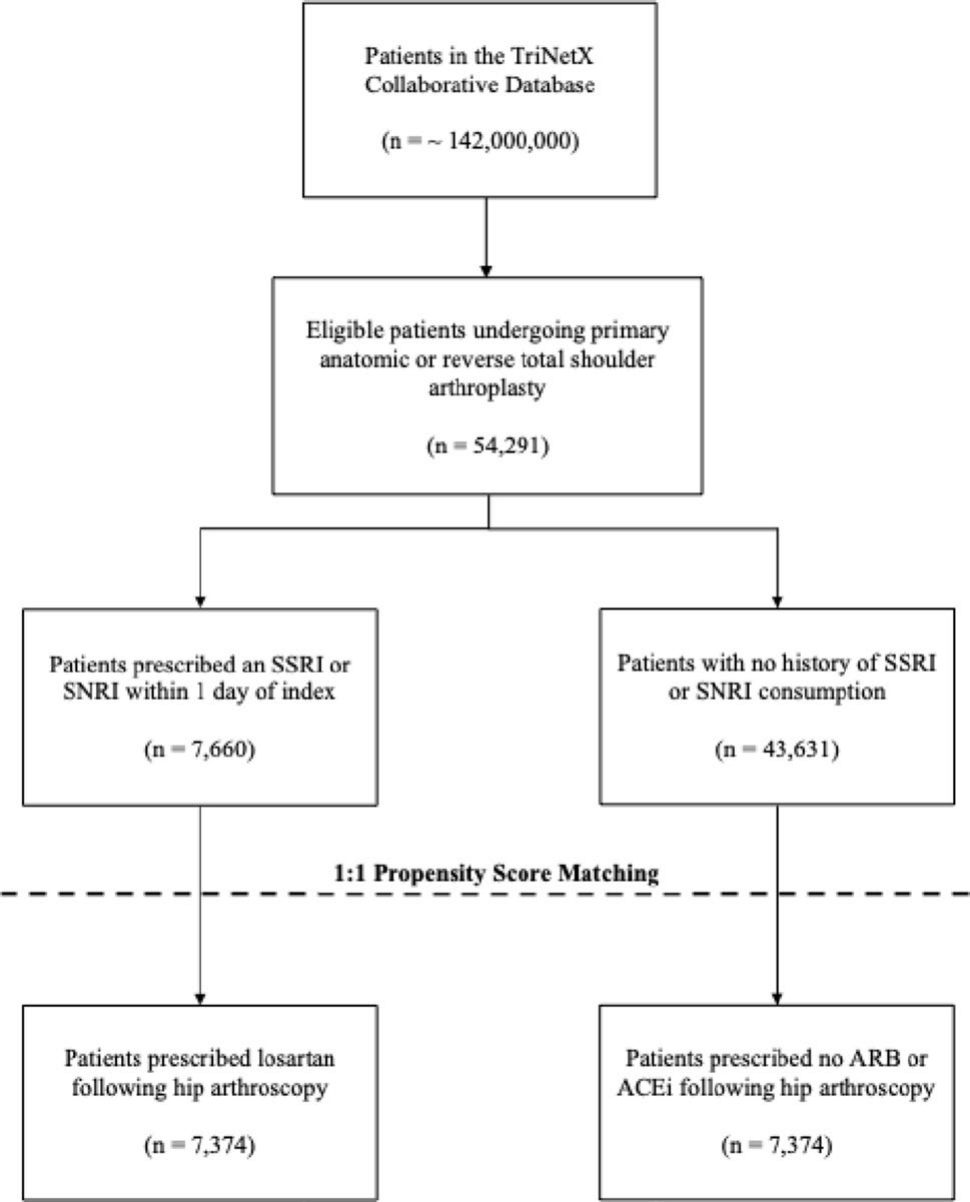
Strengthening the reporting of observational studies in epidemiology (STROBE) diagram depicting the patient selection process

A total of 54,291 who underwent TSA were identified via the TriNetX database who met the eligibility criteria. Cohort 1 contained 7,660 patients and Cohort 2 contained 43,631 patients. After the propensity match was performed, there were 7,374 patients in both Cohort 1 and Cohort 2 (Table 1) (Fig. 2). The average age of Cohort 1 and Cohort 2 was 67.7 ± 9.2 years and 67.5 ± 9.2 years, respectively (*p* = 0.131). Patients did not statistically differ across nicotine (*p* = 0.142), obesity (*p* = 0.183), diabetes (*p* = 0.643), depressive episode (*p* = 0.444), major depressive disorder (*p* = 0.296) or coagulation defects (0.161). With respect to coagulation profile, the two cohorts did not differ across percentage of patients with normal PT (10–13 s), aPTT (21–35 s) or mean platelet count. Additionally, the two cohorts did not differ for rates of prescribed anti-coagulates and anti-platelet medications (Table 1).

**Table 1:** Study cohort characteristics before and after a 1:1 propensity match

Variable	TSA + SSRI (*N* = 7,660)	TSA - SSRI (*N* = 43,631)	SMD	*P*-value	TSA + SSRI (*N* = 7,374)	TSA - SSRI (*N* = 7,374)	SMD	*P*-value
*Demographics*								
Age, years (SD)	67.7 (± 9.1)	68.5 (± 9.6)	0.091	< 0.001	67.7 (± 9.0)	67.5 (± 9.2)	0.025	0.131
White, n (%)	6,272 (82%)	34,367 (79%)	0.079	< 0.001	6,222 (84.4%)	6,261 (84.9%)	0.015	0.373
Male, n (%)	2,196 (29%)	20,514 (47%)	0.391	< 0.001	2,191 (29.7%)	2,094 (28.4%)	0.029	0.079
*Diagnoses*								
Nicotine, n (%)	286 (3.8%)	1,098 (2.2%)	0.099	< 0.001	278 (3.8%)	245 (3.3%)	0.024	0.142
Obesity, n (%)	800 (10.8%)	3,156 (6.2%)	0.164	< 0.001	779 (10.6%)	730 (9.9%)	0.022	0.183
DM, n (%)	1,124 (15%)	4,957 (9.8%)	0.163	< 0.001	1,099 (14.9%)	1,079 (14.6%)	0.008	0.643
Depressive episode, n (%)	1,335 (18%)	1,281 (2.9%)	0.508	< 0.001	1,279 (17.3%)	1,244 (16.9%)	0.013	0.444
MDD, n (%)	209 (2.8%)	357 (0.7%)	0.161	< 0.001	179 (2.4%)	160 (2.2%)	0.017	0.296
Coagulation defects, n (%)	139 (1.9%)	662 (1.3%)	0.045	< 0.001	136 (1.8%)	114 (1.5%)	0.023	0.161
*Laboratory values*								
PT (10–13 s)	1,647 (22.3%)	11,450 (22.5%)	0.01	0.708	1,647 (22.3%)	1,707 (23.1%)	0.062	0.075
aPTT (21–35 s)	1,524 (20.7%)	9,800 (19.3%)	0.089	0.002	1,524 (20.7%)	1,483 (20.1%)	0.044	0.233
Platelet count (Mean)	256 (± 74)	247 (± 69)	0.136	< 0.001	256 (± 74)	257 (± 73)	0.022	0.662
*Medications*								
Anticoagulants	354 (4.8%)	1,981 (3.9%)	0.042	< 0.001	350 (4.7%)	327 (4.4%)	0.015	0.365
Platelet aggregation inhibitors	895 (12.0%)	4,445 (8.8%)	0.108	< 0.001	871 (11.8%)	826 (11.2%)	0.019	0.246

Selective serotonin reuptake inhibitor (SSRI), diabetes mellitus (DM), major depressive disorder (MDD), prothrombin time (PT), activated partial thromboplastin time (aPTT)

### Time 0 and day 1 through 7 outcomes

A summary of the time zero and post-operative days 1–7 results can be found in Table 2. The use of SSRI and SNRIs preoperatively was associated with higher rates and odds of post-operative bleeding events and anemia on the day of operation. However, this difference was not significant. From post-operative day one through post-operative day seven, the SSRI/SNRI cohort had higher odds of hematoma/hemorrhage (OR 2.58 [95%CI 1.39–4.79], *p* = 0.002) and transfusion requirements (OR 2.78 [95%CI 1.68–4.58], *p* < 0.001). The cohorts did not differ for rates of ICU utilization (*p* = 0.053), mechanical ventilation (*p* = 0.331), post-hemorrhagic anemia (*p* = 0.227), wound dehiscence (*p* = 0.305) or shoulder irrigation and debridement (*p* = 0.531).

**Table 2:** Outcomes on the day of surgery, and from post-operative day one through post-operative day seven

Day of surgery	TSA + SSRI	TSA - SSRI	Risk difference		
Outcome	Risk (*n*, %)	Risk (*n*, %)	Per 100 (95% CI)	Odds ratio (95% CI)	*P*-value
Intraoperative hemorrhage	≤ 10 (0.1%)	≤ 10 (0.1%)	0 (-0.1, 0.1)	1.00 (0.42, 2.40)	1
Post-hemorrhagic anemia	386 (5.8%)	270 (3.9%)	1.8 (1.1, 2.6)	1.50 (1.28, 1.76)	**< 0.001**
Transfusion	28 (0.4%)	11 (0.15)	0.2 (0.1, 0.4)	2.55 (1.27, 5.13)	**0.006**

Post-operative days 1–7	TSA + SSRI	TSA - SSRI	Risk difference		
Outcome	Risk (n, %)	Risk (n, %)	Per 100 (95% CI)	Odds ratio (95% CI)	P-value
ICU	83 (1.1%)	60 (0.8%)	0.3 (0.0, 0.6)	1.39 (0.99, 1.94)	0.053
Ventilation	36 (0.5%)	29 (0.4%)	0.1 (-0.1, 0.3)	1.28 (0.78, 2.08)	0.331
Post-hemorrhagic anemia	77 (1.2%)	66 (1.0%)	0.2 (-0.1, 0.6)	1.23 (0.88, 1.71)	0.227
Hematoma/hemorrhage	36 (0.5%)	14 (0.2%)	0.3 (0.1, 0.5)	2.58 (1.39, 4.79)	**0.002**
Transfusion	58 (0.8%)	21 (0.3%)	0.5 (0.3, 0.7)	2.78 (1.68, 4.58)	**< 0.001**
Wound dehiscence	15 (0.2%)	≤ 10 (0.1%)	0.1 (-0.1, 0.2)	1.52 (0.68, 3.38)	0.305
Shoulder irrigation and debridement	13 (0.2%)	≤ 10 (0.1%)	0 (-0.1, 0.2)	1.30 (0.57, 2.97)	0.531

Bolded P-values indicate a significant difference

### Day 7 through 30 outcomes

A summary of the post-op days 7–30 outcomes can be found in Table 3. Preoperative serotonergic antidepressant use was associated with higher rates and odds of postoperative emergency department utilization (OR 1.47 [95% CI 1.20–1.78], *p* < 0.001), hospital inpatient and observatory care (OR 1.65 [95% CI 1.33–2.05], *p* < 0.001), post-hemorrhagic anemia (OR2.31 [95% CI 1.09–4.88], *p* = 0.024), hematoma/hemorrhage (OR 2.73 [95% CI 1.37–5.46], *p* = 0.003) and venous-thromboembolism (OR 2.39 [95%CI 1.33–4.30], *p* = 0.003) from post-operative day 7 through post-operative day 30.

**Table 3:** Outcomes from post-operative day seven through post-operative day 30

Post-operative days 7–30	TSA + SSRI	TSA - SSRI	Risk difference		
Outcome	Risk (*n*, %)	Risk (*n*, %)	Per 100 (95% CI)	Odds ratio (95% CI)	*P*-value
Emergency department	25 (3.5%)	174 (2.4%)	1.1 (0.5, 1.6)	1.47 (1.20, 1.78)	**< 0.001**
ICU	26 (0.4%)	21 (0.3%)	0.1 (-0.1, 0.3)	1.24 (0.70, 2.20)	0.465
Hospital inpatient	225 (3.1%)	138 (1.9%)	1.2 (0.7, 1.7)	1.65 (1.33, 2.05)	**< 0.001**
Ventilation	≤ 10 (0.1%)	≤ 10 (0.1%)	0.0 (-0.1, 0.1)	1.03 (0.43, 2.47)	0.953
Post-hemorrhagic anemia	22 (0.4%)	≤ 10 (0.1%)	0.2 (0.0, 0.4)	2.31 (1.09, 4.88)	**0.024**
Hematoma/hemorrhage	30 (0.4%)	11 (0.2%)	0.3 (0.1, 0.4)	2.73 (1.37, 5.46)	**0.003**
Transfusion	19 (0.3%)	≤ 10 (0.1%)	0.1 (0.0, 0.3)	1.90 (0.88, 4.09)	0.094
Wound dehiscence	12 (0.2%)	≤ 10 (0.1%)	0.0 (-0.1, 0.2)	1.21 (0.53, 2.81)	0.65
VTE	37 (0.6%)	16 (0.2%)	0.3 (0.1, 0.5)	2.39 (1.33, 4.30)	**0.003**
Shoulder irrigation and debridement	11 (0.2%)	≤ 10 (0.1%)	0.0 (-0.1, 0.1)	1.10 (0.47, 2.59)	0.827
Shoulder reoperation	48 (0.7%)	38 (0.5%)	0.1 (-0.1, 0.4)	1.27 (0.83, 1.94)	0.279
Mortality	≤ 10 (0.1%)	≤ 10 (0.1%)	0.0 (-0.1, 0.1)	1.00 (0.42, 2.40)	1

Bolded P-values indicate a significant difference

### Day 30 through 90 outcomes

A summary of the outcomes from 30-day to 90-day follow-up can be found in Table 4. Preoperative SSRI/SNRI use was associated with higher rates and odds of emergency department utilization (OR 1.28 [95%CI 1.10–1.49], *p* = 0.002), ICU requirement (OR 1.96 [95%CI 1.14–3.36], *p* = 0.13) and hospital inpatient and observatory care (OR 1.33 [95%CI 1.07–1.64], *p* = 0.009). Cohorts did not differ across odds for post-hemorrhagic anemia, hematoma/hemorrhage, transfusion, wound dehiscence, shoulder reoperations and shoulder irrigation and debridement.

**Table 4:** Outcomes from post-operative day 30 through post-operative day 90

Post-operative days 30–90	TSA + SSRI	TSA - SSRI	Risk difference		
Outcome	Risk (*n*, %)	Risk (*n*, %)	Per 100 (95% CI)	Odds ratio (95% CI)	*P*-value
Emergency department	385 (5.2%)	304 (4.1%)	1.1 (0.4, 1.8)	1.28 (1.10, 1.49)	**0.002**
ICU	39 (0.5%)	20 (0.3%)	0.3 (0.1, 0.5)	1.96 (1.14, 3.36)	**0.013**
Hospital inpatient	204 (2.8%)	155 (2.1%)	0.7 (0.2, 1.2)	1.33 (1.07, 1.64)	**0.009**
Ventilation	≤ 10 (0.1%)	≤ 10 (0.1%)	0.0 (-0.1, 0.1)	1.03 (0.43, 2.47)	0.953
Post-hemorrhagic anemia	20 (0.3%)	15 (0.2%)	0.1 (-0.1 0.3)	1.40 (0.72, 2.73)	0.321
Hematoma/hemorrhage	13 (0.2%)	≤ 10 (0.1%)	0.0 (-0.1, 0.2)	1.30 (0.57, 2.96)	0.531
Transfusion	11 (0.1%)	12 (0.2%)	0.0 (-0.1, 0.1)	0.92 (0.40, 2.08)	0.835
Wound dehiscence	≤ 10 (0.1%)	≤ 10 (0.1%)	0.0 (-0.1, 0.1)	1.01 (0.42, 2.43)	0.982
VTE	21 (0.3%)	14 (0.2%)	0.1 (-0.1, 0.3)	1.55 (0.85, 2.81)	0.149
Shoulder reoperation	65 (0.9%)	51 (0.7%)	0.2 (-0.1, 0.5)	1.28 (0.88, 1.85)	0.192
Shoulder irrigation and debridement	≤ 10 (0.1%)	≤ 10 (0.1%)	0.0 (-0.1, 0.1)	1.00 (0.42, 2.40)	1
Mortality	14 (0.2%)	≤ 10 (0.1%)	0.1 (-0.1, 0.2)	1.40 (0.62, 3.15)	0.414

Bolded P-values indicate a significant difference

## Discussion

Our propensity-matched analysis of a large international database demonstrated that SSRIs and SNRIs were associated with higher rates of acute bleeding events in the short-term post-operative period. Within the first week of surgery, patients on SSRI/SNRIs were at increased odds for hematoma/hemorrhage and ultimately transfusion of blood product. From day seven onward, these patients continued to be at higher risk for post-hemorrhagic anemia and hematoma/hemorrhage and now were found to have higher rates for ED utilization, hospital inpatient and observatory care and venous thromboembolism. From 30 to 90 days, patients on SSRI/SNRIs continued to exhibit higher rates of ED, ICU, and hospital use but no longer differed across the hemostasis parameters.

Serotonergic antidepressants (e.g., SSRIs, SNRIs) are commonly prescribed medications that are used to treat numerous psychiatric conditions [15–20]. Although they are considered safe medications with relatively wider therapeutic indices, the serotonergic antidepressants have been associated with an increased risk of bleeding [22–25]. Serotonin is a known platelet activator and vasoconstrictive molecule. SSRIs and SNRIs inhibit the reuptake of serotonin mainly in the central nervous system. However, their effect is not limited to the central nervous system, and they can inhibit serotonin reuptake elsewhere. When platelet serotonin transporter is inhibited, platelet activation and aggregation are all decreased, which in turn leads to increased bleeding [33, 34].

Prior studies have demonstrated an association between serotonergic antidepressants and bleeding episodes following orthopaedic surgery. In a case control study of 1,539 patients undergoing elective spinal fusion, Sayadiour et al. showed that patients on serotonergic antidepressants had significantly larger volumes of blood loss and longer lengths of hospital stay when compared to a matched control group [27]. After controlling for potentially confounding variables, Belay et al. revealed that SSRI use was associated with increased blood loss following TKA and total hip arthroplasty (THA) and that SSRI utilization was predictive of future transfusion [32]. Finally, Bourget-Murray et al. similarly showed that SSRI use prior to elective TKA and THA was associated with inferior postoperative functional outcomes as well as increased transfusion rates, lengths of stay, readmissions, and “medical events” (e.g., MI, VTE, stroke, ileus, gastrointestinal bleeding, pneumonia) [35]. Although we did not observe increased rates of MI, stroke, or other medical complications, our findings are largely similar. Overall, patients within our current study were at higher risk for acute bleeding events through 30-days post-operatively. Importantly, these outcomes were seen even when controlling for PT, aPTT, platelet count, anti-coagulant prescription medications, anti-platelet prescription medications and coagulopathies. Further studies are required to confirm these additional findings. Considering these findings, we recommend a multi-disciplinary discussion between the orthopaedic surgeon, primary care provider, and/or treating psychiatrist to determine if serotonergic antidepressants should be temporarily held prior to shoulder arthroplasty, especially for patients at an already higher risk for bleeding events. Of course, any discussion should weigh the risks, benefits, and alternatives to discontinuing these medications.

Numerous studies have demonstrated that anxiety, depression, and other psychiatric illnesses predict poor outcomes after orthopaedic surgery [36–48]. In the current study, we propensity matched for depressive episode and major depressive disorder, although we did not correlate outcomes with the presence of a preoperative psychiatric illness. While the study focuses on the outcomes of acute bleeding events in the form of anemia, hematoma/hemorrhage and transfusions, underlying psychiatric comorbidities may play a role in ED visits, hospital inpatient services and ICU services.

The results of this study must be interpreted within its limitations. TriNetX retrospectively collects data from the electronic medical records (EMR) of participating healthcare organizations. As with any data collected in this fashion, data may be incomplete and diagnoses, procedures, and clinical events may be coded incorrectly. Additionally, events and diagnoses may be underreported as ICD and CPT codes may not be sensitive enough to capture all events. Moreover, rounding events with counts < 10 to 10 may overestimate the risk of these outcomes. Another limitation of the TriNetX database is that complications and postoperative events cannot be linked to the index procedure with absolute certainty. For example, in the hypothetical scenario where a patient underwent TSA (CPT 23472) but had a history of prior TAA (CPT 27702), the occurrence of PJI (e.g., ICD-10-CM code T84.59 “infection and inflammatory reaction due to other internal joint prosthesis”) cannot be definitively connected to the either the TSA or the TAA in the TriNetX database. Therefore, we excluded patients with a prior history of non-shoulder joint arthroplasty to reduce this potential confounding effect. Ultimately, this decreased the size of our study population and the number of postoperative events but increased the validity of our results. Despite propensity matching, our data may not account for all co-morbidities that may potentially lead to increased risk for acute bleeding events and health-care utilization. Finally, surveillance bias may have emerged due to a greater propensity for SSRI/SNRI users to utilize healthcare services, including ED visits, ICU visits, and inpatient admission. This may have also contributed to detection bias with inflated detection of VTE and bleeding due to greater healthcare utilization. Future analyses may include sensitivity checks that restrict outcomes to inpatient-coded events or exclude diagnoses made in ED visits.

The strengths of our study include the large size of our cohorts, the propensity-matched nature of our study design, and specific focus on short-term bleeding events following TSA.

## Conclusion

SSRIs/SNRIs appear to acutely increase risk for post-operative bleeding events, but do not appear to affect long term risk. Perioperative administration of SSRI’s/SNRI’s during elective TSA requires individualized, multidisciplinary decision making that balances psychiatric benefits with the potential for bleeding events, particularly in high risk patients.

## Supplementary Information

Below is the link to the electronic supplementary material.

Supplementary Material 1

Supplementary Material 2

Supplementary Material 3

## Data Availability

The data that support the findings of this study are derived from the TriNetX Network but are not publicly available due to data use agreements and patient privacy constraints.
