# Supplementary material for: Serotonergic antidepressants are associated with increased bleeding events within 30-days after total shoulder arthroplasty: a propensity-matched analysis of 54,291 patients
Source: Arch Orthop Trauma Surg. 2026 Mar 12;146(1):111. doi: 10.1007/s00402-026-06254-y (PMC12982205; doi:10.1007/s00402-026-06254-y)
Supplement: Supplementary file 1 — Supplementary Material 1 [file 402_2026_6254_MOESM1_ESM.docx]

**Supplemental table 1.** Codes used for the creation of the TSA with SSRI use cohort. TriNetX utilizes a conversion algorithm to code ICD-9 codes under their ICD-10 counterparts.

| **TSA with SSRI use** | | |
| --- | --- | --- |
|  | Code | Description |
| **Must Have below between May 14th 2012 - May 14th 2022** | | |
|  | CPT 23472 | Arthrodesis, ankle, open |
| **Must include below within 1 day of CPT 23472** | | |
|  |  | citalopram |
| or |  | escitalopram |
| or |  | fluoxetine |
| or |  | fluvoxamine |
| or |  | paroxetine |
| or |  | sertraline |
| or |  | venlafaxine |
| or |  | desvenlafaxine |
| or |  | duloxetine |
| or |  | milnacipran |
| or |  | levomilnacipran |
| **Cannot include below within 1 month prior to CPT 23472** | | |
|  | CPT 1005073 | Arthroplasty, knee, condyle and plateau |
| or | ICD10 S42.29 | Other fracture of upper end of humerus |
| or | ICD10 S42.26 | Fracture of lesser tuberosity of humerus |
| or | ICD10 S42.25 | Fracture of greater tuberosity of humerus |
| or | ICD S42.24 | 4-part fracture of surgical neck of humerus |
| or | ICD10 S42.23 | 3-part fracture of surgical neck of humerus |
| or | ICD10 S42.22 | 2-part fracture of surgical neck of humerus |
| or | ICD10 S42.2 | Fracture of upper end of humerus |
| or | CPT 1004348 | Arthroplasty, elbow |
| or | CPT 1014080 | Arthroplasty, radial head |
| or | CPT 1004746 | Arthroplasty, metacarpophalangeal joint |
| or | CPT 1004749 | Arthroplasty, interphalangeal joint |
| or | CPT 1014588 | Arthroplasty, ankle |
| or | CPT 1004749 | Arthroplasty, interphalangeal joint |
| or | CPT 1005065 | Arthroplasty, patella |
| or | CPT 25447 | Arthroplasty, interposition, intercarpal or carpometacarpal joints |
| or | CPT 23470 | Arthroplasty, glenohumeral joint; hemiarthroplasty |
| or | CPT 27125 | Hemiarthroplasty, hip, partial (eg, femoral stem prosthesis, bipolar arthroplasty) |
| or | CPT 1004746 | Arthroplasty, metacarpophalangeal joint |
| or | ICD10 T84.53 | Infection and inflammatory reaction due to internal right knee prosthesis |
| or | CPT 27130 | Arthroplasty, acetabular and proximal femoral prosthetic replacement (total hip arthroplasty), with or without autograft or allograft |
| or | ICD10 T84.54 | Infection and inflammatory reaction due to internal left knee prosthesis |
| or | ICD10 T84.51 | Infection and inflammatory reaction due to internal right hip prosthesis |
| or | ICD10 T84.52 | Infection and inflammatory reaction due to internal left hip prosthesis |
