# Supplementary material for: Serotonergic antidepressants are associated with increased bleeding events within 30-days after total shoulder arthroplasty: a propensity-matched analysis of 54,291 patients
Source: Arch Orthop Trauma Surg. 2026 Mar 12;146(1):111. doi: 10.1007/s00402-026-06254-y (PMC12982205; doi:10.1007/s00402-026-06254-y)
Supplement: Supplementary file 3 — Supplementary Material 3 [file 402_2026_6254_MOESM3_ESM.docx]

**Supplemental Table 3.** Codes used for outcome measures including descriptions of the CPT/ICD codes. TriNetX utilizes a conversion algorithm to code ICD-9 codes under their ICD-10 counterparts.

| **Outcome definition codes** | | | |
| --- | --- | --- | --- |
|  |  | Codes | Description |
| Emergency Department | | | |
|  |  | CPT 1013711 | Emergency Department Services |
| Hospital Inpatient | | | |
|  |  | CPT 1013659 | Hospital Inpatient and Observation Care Services |
| Critical Care | | | |
|  |  | CPT 1013729 | Critical Care Services |
| Intraoperative hemorrhage | | | |
|  |  | ICD10 M96.81 | Intraoperative hemorrhage and hematoma of a musculoskeletal structure complicating a procedure |
| Hematoma/Hemorrhage | | | |
|  |  | ICD10 M96.84 | Postprocedural hematoma and seroma of a musculoskeletal structure following a procedure |
| or |  | ICD10 M96.83 | Postprocedural hemorrhage of a musculoskeletal structure following a procedure |
| or |  | ICD10 T84.83 | Hemorrhage due to internal orthopedic prosthetic devices, implants and grafts |
| or |  | CPT 23030 | Incision and drainage, shoulder area; deep abscess or hematoma |
| or |  | ICD10 M79.81 | Nontraumatic hematoma of soft tissue |
| or |  | ICD10 L76.32 | Postprocedural hematoma of skin and subcutaneous tissue following other procedure |
| or |  | ICD10 L76.34 | Postprocedural seroma of skin and subcutaneous tissue following other procedure |
| Post-hemorrhagic anemia | | | |
|  |  | ICD10 D62 | Acute posthemorrhagic anemia |
| Transfusion |  |  |  |
|  |  | CPT 36430 | Transfusion, blood or blood components |
| Venous Thromboembolism | | | |
|  |  | ICD10 I26 | Pulmonary embolism |
| or |  | ICD10 I82.4 | Acute embolism and thrombosis of deep veins of lower extremity |
| or |  | ICD10 I82.6 | Acute embolism and thrombosis of veins of upper extremity |
| Wound Dehiscence | | | |
|  |  | ICD10 T81.3 | Disruption of wound, not elsewhere classified |
| Ventilation | | | |
|  |  | ICD10 5A09357 | Assistance with Respiratory Ventilation, Less than 24 Consecutive Hours, Continuous Positive Airway Pressure |
| or |  | ICD10 5A09457 | Assistance with Respiratory Ventilation, 24-96 Consecutive Hours, Continuous Positive Airway Pressure |
| or |  | ICD10 5A09557 | Assistance with Respiratory Ventilation, Greater than 96 Consecutive Hours, Continuous Positive Airway Pressure |
| or |  | ICD10 5A1945Z | Respiratory Ventilation, 24-96 Consecutive Hours |
| or |  | ICD10 5A1955Z | Respiratory Ventilation, Greater than 96 Consecutive Hours |
| or |  | ICD10 5A1935Z | Respiratory Ventilation, Less than 24 Consecutive Hours |
| Shoulder Irrigation and Debridement | | | |
|  |  | CPT 23030 | Incision and drainage, shoulder area; deep abscess or hematoma |
| Shoulder Reoperation | | | |
|  |  | CPT 23105 | Arthrotomy; glenohumeral joint, with synovectomy, with or without biopsy |
| or |  | CPT 23107 | Arthrotomy, glenohumeral joint, with joint exploration, with or without removal of loose or foreign body |
| or |  | CPT 23334 | Removal of prosthesis, includes debridement and synovectomy when performed; humeral or glenoid component |
| or |  | CPT 23335 | Removal of prosthesis, includes debridement and synovectomy when performed; humeral and glenoid components (eg, total shoulder) |
| or |  | CPT 23473 | Revision of total shoulder arthroplasty, including allograft when performed; humeral or glenoid component |
| or |  | CPT 23474 | Revision of total shoulder arthroplasty, including allograft when performed; humeral and glenoid component |
| or |  | CPT 11981 | Insertion, drug-delivery implant (ie, bioresorbable, biodegradable, non-biodegradable) |
| or |  | CPT 29821 | Arthroscopy, shoulder, surgical; synovectomy, complete |
| or |  | CPT 29820 | Arthroscopy, shoulder, surgical; synovectomy, partial |
| or |  | CPT 29822 | Arthroscopy, shoulder, surgical; debridement, limited, 1 or 2 discrete structures (eg, humeral bone, humeral articular cartilage, glenoid bone, glenoid articular cartilage, biceps tendon, biceps anchor complex, labrum, articular capsule, articular side of the rotator cuff, bursal side of the rotator cuff, subacromial bursa, foreign body[ies]) |
| or |  | CPT 29823 | Arthroscopy, shoulder, surgical; debridement, extensive, 3 or more discrete structures (eg, humeral bone, humeral articular cartilage, glenoid bone, glenoid articular cartilage, biceps tendon, biceps anchor complex, labrum, articular capsule, articular side of the rotator cuff, bursal side of the rotator cuff, subacromial bursa, foreign body[ies]) |
| or |  | CPT 29825 | Arthroscopy, shoulder, surgical; with lysis and resection of adhesions, with or without manipulation |
| or |  | CPT 23030 | Incision and drainage, shoulder area; deep abscess or hematoma |
| or |  | CPT 23040 | Arthrotomy, glenohumeral joint, including exploration, drainage, or removal of foreign body |
